# Computational Insights into Dion–Jacobson Type Oxide Ion Conductors

**DOI:** 10.1021/acs.jpcc.4c01166

**Published:** 2024-05-24

**Authors:** Bettina Schwaighofer, Miguel Angel Gonzalez, Ivana Radosavljevic Evans

**Affiliations:** † Institut Laue Langevin, 71 Rue de Martyrs, Grenoble 38000, France; ‡ Department of Chemistry, Durham University, Science Site, South Road, Durham DH1 3LE, U.K.

## Abstract

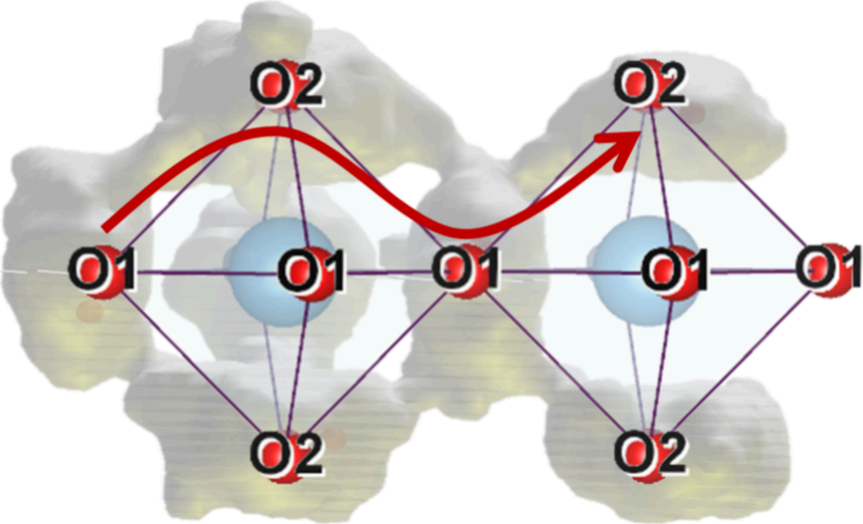

Dion–Jacobson type materials have recently emerged as a new structural family of oxide ion conductors, materials important for applications in a variety of electrochemical devices. While some attempts to improve their ionic conductivity have been reported, a detailed understanding of the underlying oxide ion diffusion mechanisms in these materials is still missing. To explore the structure–property relationships leading to the favorable properties, we carried out *ab initio* molecular dynamics simulations of oxide ion diffusion in CsBi_2_Ti_2_NbO_10−δ_. Our computational study reveals significant out-of-plane dynamics, indicating that the dominant pathway for oxide ion migration is via jumps into and out of the (*ab*) crystallographic plane. This suggests that further improvement of oxide ion conductivity relative to CsBi_2_Ti_2_NbO_10−δ_ could be achieved by enhancing the rotational flexibility of the coordination polyhedra located in the inner perovskite layer, thereby facilitating faster out-of-plane motions.

## 1 Introduction

Increasing interest in oxide ion conductors is fostered by their potential applications in energy materials, especially in solid oxide fuel and electrolyte cells, but also as oxygen sensors and oxygen permeable membranes. However, current materials require very high temperatures to achieve a sufficiently high ionic conductivity.^1−3^ The discovery of high ionic conductivity in less-explored structure types provides significant opportunities for improving physical properties by chemical modifications, potentially giving rise to new, better-performing oxide ion conductors.

Atomic-level understanding of the migration pathways of oxide ions in solid state materials can facilitate the development of new oxide ion conductors. Atomistic simulations have long been an established computational method used for this purpose.^4,5^ However, more recent discoveries of high oxide ion conductivity in structurally more complex compounds, in which the ionic diffusion involves the breaking and making of chemical bonds, meant that *ab initio* molecular dynamics (AIMD) simulations were necessary to accurately model the properties of these materials. The challenge in this approach has traditionally been the computational cost related to the size of the simulation box, which can realistically be probed. Nevertheless, AIMD simulations have successfully been used to elucidate ionic conduction pathways and mechanisms in oxide ion conductors belonging to a number of different structural families, including fluorites,^6−9^ apatites,^10,11^ LAMOX,^12^ and perovskite-related^13−16^ materials.

**Figure 1:**
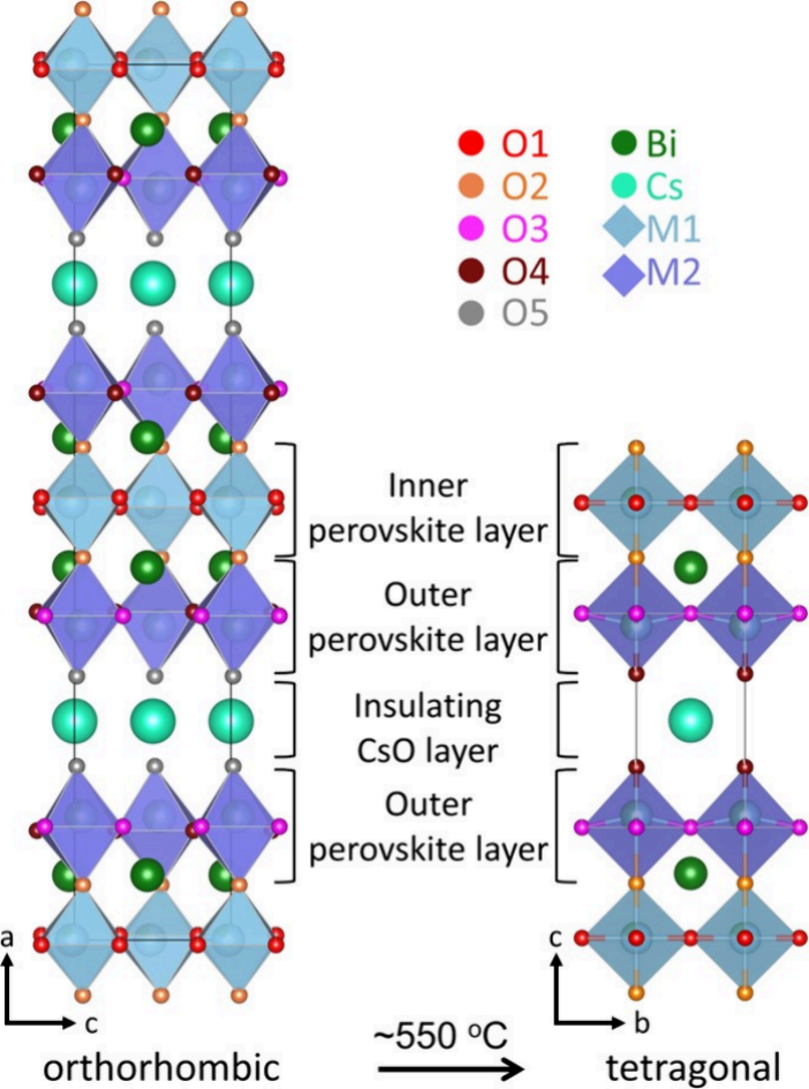
Neutron diffraction-derived structure of CsBi_2_Ti_2_NbO_10−δ_: low-temperature, orthorhombic phase (left), and high-temperature, tetragonal phase (right).^17^

The first report of significant oxide ion conductivity in a compound crystallizing in the Dion–Jacobson phase was published in 2020; CsBi_2_Ti_2_NbO_9.8_ exhibits a bulk ionic conductivity of 1.5 × 10^–2^ S cm^–^^1^ at 600 °C and 8.9 × 10^–2^ S cm^–^^1^ at 800 °C, with oxide ions being the dominant charge carriers.^17^ The structure of CsBi_2_Ti_2_NbO_10−δ_ can be described as layered and perovskite-like (Figure 1). The inner and outer perovskite layers (light- and dark-blue polyhedra, respectively) consist of cubically arranged MO_6_ octahedra (M = Ti^4+^/Nb^5+^), with Bi^3+^ cations occupying the central 12-coordinate sites of these cuboides.^17,18^ The perovskite layers are separated by large Cs^+^ cations located in the insulating layer, limiting the perovskite-type blocks in the *c*-direction. Between 540 and 560 °C, CsBi_2_Ti_2_NbO_10−δ_ undergoes a reversible first-order phase transition from the low-temperature orthorhombic *Ima2* structure to a tetragonal *P4/mmm* one, leading to an abrupt increase of oxide ion conductivity by approximately 1 order of magnitude.^17,19^ At room temperature, there are two crystallographically distinct M sites; the occupancy of M1 is Ti_0.822(2)_Nb_0.178(2)_ and that of M2 Ti_0.589(1)_Nb_0.411(1)_.^17^ Of the five unique oxygen sites, O2 and O3 are fully occupied, while vacancies are distributed over O1 (0.994(2)), O4 (0.998(2)), and O5 (0.995(2)) sites.^17^

In the tetragonal phase at 700 °C, the M1 and M2 site occupancies are slightly changed (Ti_0.804(2)_Nb_0.196(2)_ for M1, Ti_0.598(1)_Nb_0.402(1)_ for M2). The oxygen atom arrangement differs more substantially from that at room temperature, with only four unique sites present: O1 with essentially unchanged fractional occupancy, and O2, O3, and O4 with occupancies decreased to 0.976(3), 0.991(1), and 0.944(3), respectively.^17^ It was suggested that this reversible decrease in oxygen content contributes to the high oxide ion diffusion in CsBi_2_Ti_2_NbO_10−δ_ by vacancy hopping via O1 and O2 sites.^17^

On this basis, several attempts to improve the properties of CsBi_2_Ti_2_NbO_10−δ_ by introducing more vacancies into the structure have been reported, for example, the CsBi_2–*x*_M_*x*_Ti_2_NbO_10–*x*/2_ (M = Mg^2+^, Ca^2+^, Sr^2+^, Ba^2+^) series.^20^ However, among these materials, only CsBi_1.9_Sr_0.1_Ti_2_NbO_9.95_ demonstrated a moderate improvement of the total measured conductivity relative to the parent system (0.7 × 10^–3^ S cm^–1^ vs 0.4 × 10^–3^ S cm^–1^ at 600 °C, and 6 × 10^–3^ S cm^–1^ vs 3 × 10^–3^ S cm^–1^ at 800 °C).^20^ Other chemical modifications, which retained oxide ion vacancies, such as the CsA_2_Ti_2_NbO_10−δ_ (A = La^3+^, Pr^3+^, Nd^3+^, Sm^3+^) materials, also resulted in deterioration of properties; for example, the best performer in this series, CsLa_2_Ti_2_NbO_10−δ_, exhibits a bulk conductivity of 2.2 × 10^–4^ S cm^–1^ at 800 °C, which is significantly lower than the bulk conductivity of CsBi_2_Ti_2_NbO_10−δ_.^21^ Furthermore, introduction of interstitial oxide ions by varying the Ti^4+^/Nb^5+^ ratio resulted in slightly increased total conductivity compared to CsBi_2_Ti_2_NbO_10−δ_, with the best performance exhibited by CsBi_2_Ti_1.8_Nb_1.2_O_10.10_.^22^

These studies demonstrate that, while there is significant potential for chemical modifications of Dion–Jacobson type oxide ion conductors with both vacancies and interstitial defects, an in-depth understanding of ionic diffusion mechanisms, necessary for systematic and more significant improvements of properties of these materials, is still missing. Indeed, a recent comprehensive review identified *ab initio* molecular dynamics simulations as a crucial step in providing such atomic-level insight.^22^

Here, we report *ab initio* molecular dynamics (AIMD) calculations of oxide ion diffusion in this new structural family of ionic conductors. Using the composition CsBi_2_Ti_2_NbO_10−δ_, very long (300 ps) simulations were performed at five different temperatures. These allowed observation of continuous oxide ion diffusion pathways, while a detailed analysis of the simulations revealed that jumps out of the (*ab*) plane in the *c* direction are significantly more frequent than direct O1–O1 jumps. This suggests that long-range diffusion occurs via an O1–O2–O1 diffusion pathway, highlighting the importance of rotational flexibility of the coordination polyhedra centered on the M1 metal sites to a high ionic conductivity, thereby demonstrating how a more targeted modification of the oxide ion conductivity is possible.

## 2 Computational Methods

The Vienna *ab initio* simulation package (VASP) code was used to perform *ab initio* molecular dynamics (AIMD) simulations based on density functional theory (DFT).^23^ All simulations were carried out using the GGA-PBE (Perdew–Burke–Ernzerhof generalized-gradient-approximation)^24^ exchange-correlation functional and the projector augmented wave (PAW) method,^25^ in combination with the GW approximation to obtain the electronic structure.^26^

The conducting high-temperature structure given by Zhang et al.^17^ was used to create a 4 × 4 × 1 (15.57613 × 15.57613 × 15.58224 Å) near-isotropic supercell of CsBi_2_Ti_2_NbO_10−δ_ containing 252 atoms in total. Nb and Ti atoms were randomly placed on M1 and M2 sites corresponding to their relative occupancies determined from neutron diffraction. Then, four random oxygen vacancies were created to account for the known oxygen deficiency, resulting in a simulation box of composition Cs_16_Bi_32_Ti_32_Nb_16_O_156_, corresponding to the formula CsBi_2_Ti_2_NbO_9.75_, which is very close to the actual composition determined from neutron diffraction data at 700 °C, i.e., CsBi_2_Ti_2_NbO_9.8_. To study the influence of the distribution of vacancies on the simulations, three independent models were created. In model 1, one vacancy is introduced on each of the O2 and O3 sites and two vacancies on O4 sites, while all O1 sites are occupied. The second model contains one vacancy on each of the different types of oxygen sites (O1, O2, O3, and O4). Finally, in model 3, the O3 and O4 sites are fully occupied, while the O1 and O2 sites contain two vacancies each.

First, the three models were simulated at 1000 °C for 100 ps and then, based on the analysis presented in Section 3.1, model 2 was selected for further simulations at the following temperatures: 600, 800, 1000, 1200, and 1400 °C. A cutoff energy of 300 eV and a time-step of 0.002 ps were used in all the AIMD calculations, which extended for at least 150,000 steps (i.e., 300 ps). The generated trajectories were analyzed with MDANSE^27^ and the cloud plots showing the oxygen diffusion pathways produced with LAMP.^28^

## 3 Results and Discussion

### 3.1 Influence of the Initial Vacancy Distribution on Simulated Oxygen Diffusion in CsBi_2_Ti_2_NbO_10−δ_

**Figure 2:**
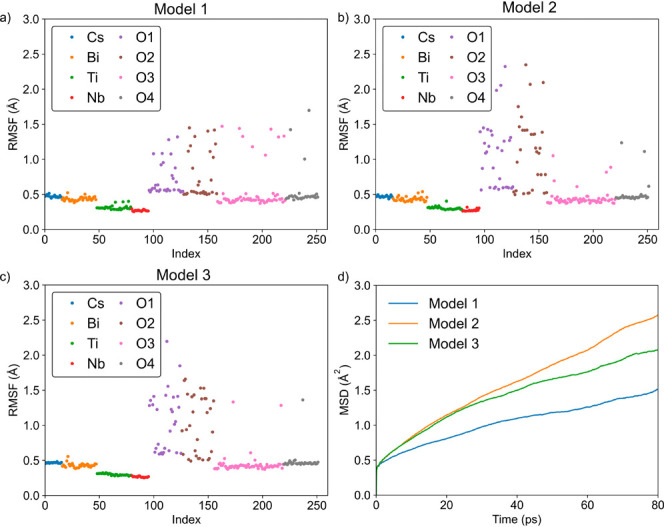
Root-mean-square fluctuation (RMSF) of each atom in the simulation box after 100 ps at 1000 °C for three different initial models containing four vacancies each: model 1 (one vacancy in O2 and O3 and two in O4) (a), model 2 (one vacancy in O1, O2, O3, and O4) (b), and model 3 (two vacancies in O1 and two in O2) (c); oxygen mean square displacements of the three models (d).

Because of the abrupt increase in oxide ion conductivity at the phase transition temperature, which coincides with a reversible decrease in oxygen content, it has been proposed that the intrinsic oxygen vacancies are a key factor contributing to the high conductivity of the tetragonal phase of CsBi_2_Ti_2_NbO_10−δ_.^17^ Therefore, the impact of the initial distribution of vacancies was explored by comparing the root-mean-square fluctuation (RMSF) of all atoms during the simulations performed using the three models described in Section 2. The RMSF measures how much on average an atom moves away from its initial position, so especially mobile atoms can be easily identified. The results are displayed in Figure 2.

Despite the presence of two vacancies on the O4 site in model 1, the RMSF of oxygen atoms located on this site is low (gray dots, Figure 2a), and oxygen atoms on O1 and O2 sites (purple and brown dots, Figure 2a) are the most mobile. Model 2 indicates increased dynamics on O1 and O2 sites (purple and brown dots, Figure 2b) located in the inner perovskite layer, compared to O3 and O4 (pink and gray dots, Figure 2b), which are located in the outer perovskite layer. The presence of extra vacancies on the O1 and O2 sites (model 3) does not affect the RMSF significantly compared to the model containing one vacancy on each site (Figure 2b,c). Finally, the comparison of the mean square displacement (MSD) curves for oxygen, shown in Figure 2d, indicates that model 2 results in the fastest dynamics.

These MSD curves can be used to get an estimate of the oxygen diffusion coefficient (*D*) from their long-time slope. The values of *D* for models 1, 2, and 3 are 2.1 ± 0.5 × 10^–7^, 4.4 ± 0.4 × 10^–7^, and 3.3 ± 0.9 × 10^–7^ cm^2^ s^–1^, respectively. While the statistical uncertainties do not allow to discriminate definitely between models 2 and 3, these self-diffusion coefficients together with the behavior of the MSD curves clearly show that enhancing the number of vacancies on the O1 and O2 sites in the starting model does not increase the number of jumps observed during the simulation significantly. As model 2 demonstrates the highest MSD while not being biased with respect to the vacancy distribution and thereby increasing the probability of observing specific jumps, this model was user for additional, more detailed simulations.

### 3.2 Temperature Dependence of the Oxide Ion Dynamics in CsBi_2_Ti_2_NbO_10−δ_ from AIMD

**Figure 3:**
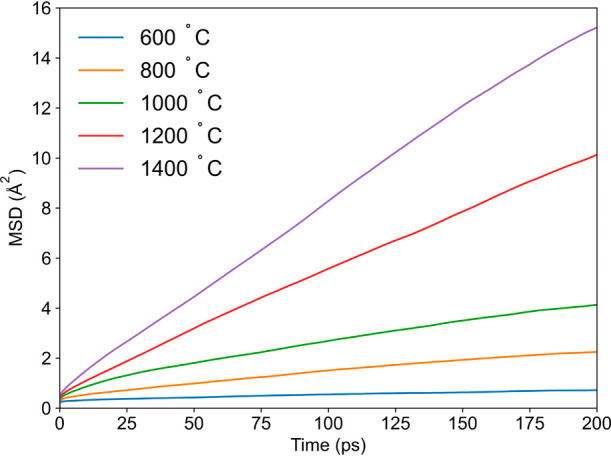
Oxygen mean square displacement curves derived from the AIMD simulations at 600, 800, 1000, 1200, and 1400 °C.

Five longer AIMD runs covering a temperature range between 600 and 1400 °C were performed using model 2. Figure 3 shows the corresponding MSD of the oxygen atoms at each temperature.

**Figure 4:**
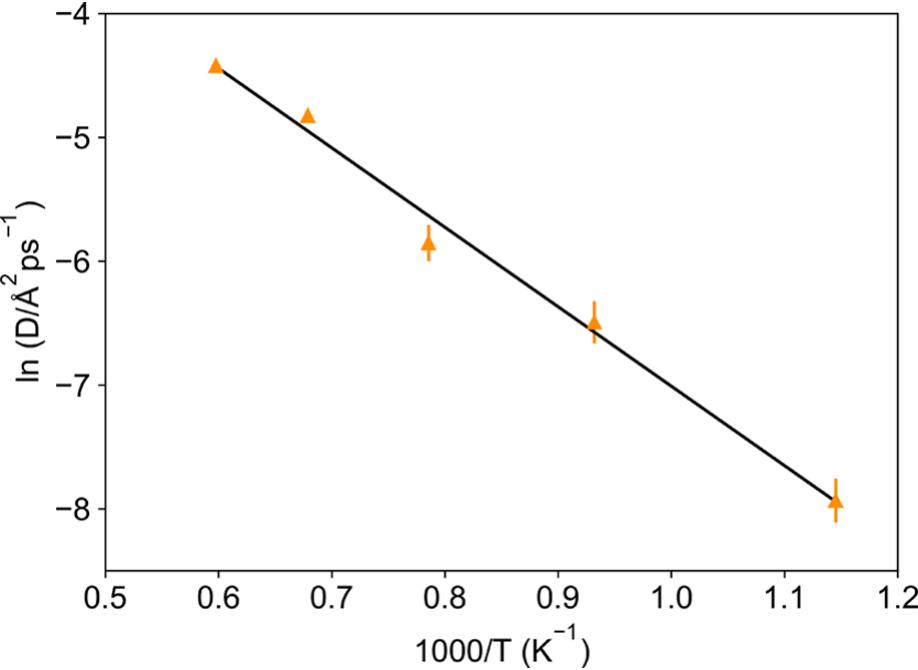
Temperature dependence of the self-diffusion coefficient (*D*) derived from the oxygen MSD and fit (black line) to an Arrhenius law.

As expected, the temperature increase significantly enhances oxide ion dynamics causing a systematic rise in the MSD. From the slope of the MSD curves of all oxygen atoms, the diffusion coefficient (*D*) was obtained per the Einstein relation, which then allows determination of the activation energy for diffusion through an Arrhenius type relationship (Figure 4).

The obtained diffusion coefficients are 0.35 ± 0.06 × 10^–7^ (600 °C), 1.5 ± 0.3 × 10^–7^ (800 °C), 2.9 ± 0.4 × 10^–7^ (1000 °C), 8.0 ± 0.3 × 10^–7^ (1200 °C), and 12.0 ± 0.3 × 10^–7^ (1400 °C) cm^2^ s^–1^. The pre-exponential factor is 9 ± 3 × 10^–6^ cm^2^ s^–1^, and the activation energy derived from the fit of the *D* values is 0.55 ± 0.03 eV, slightly lower than the activation energy obtained from impedance data (0.846 ± 0.005 eV).^4^ However, such differences between microscopic measurements like NMR and AIMD, probing dynamics on the atomic level, and macroscopic measurements like impedance spectroscopy, which are affected by the sample pellet density and morphology, are common in the literature.^29−36^

**Figure 5:**
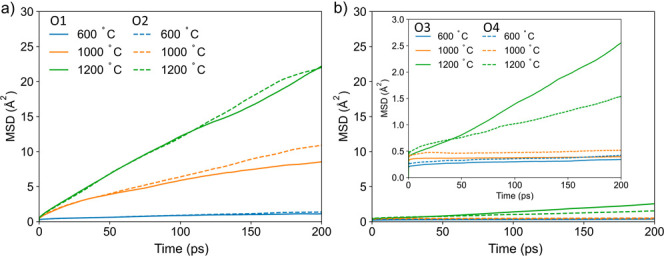
Mean square displacement at 600, 1000, and 1200 °C for O1 and O2 (a) and O3 and O4 (b) oxygen atoms. The curves are shown on the same scale to evidence the difference between (O1, O2) and (O3, O4) atoms. The inset displays the MSDs for O3 and O4 on a smaller scale to show their behavior more clearly.

Figure 5 provides further insight on the oxygen dynamics by showing separately the MSD of the oxygen atoms initially placed at different crystallographic sites. It is apparent that the mobility of oxygen atoms occupying the O1 and O2 sites (Figure 5a) is considerably larger than that of the atoms at O3 and O4 sites (Figure 5b). At 1200 °C, the MSD of O1 and O2 atoms at t = 200 ps is approximately 22 Å^2^, suggesting that each oxygen atom on these two crystallographic sites has moved by approximately 4.7 Å. This is larger than the maximum distance between oxygen sites on a Ti/NbO_6_ unit of ∼3.9 Å, indicating that long-range diffusion of oxide ions is captured by the simulations. It should also be noted that the classification of the MSD curves in Figure 5 is based on the initial position of the oxygen atoms. However, at high temperatures, the enhanced dynamics of the oxide ions results in a fast exchange between both sites, blurring the distinction between O1 and O2 atoms and resulting in similar MSD curves (Figure 5a). In contrast to that, the MSD of O3 and O4 oxygen atoms (Figure 5b) are very small. In fact, even at 1000 °C, the MSD of O3 and O4 atoms after 200 ps is only about 0.5 Å^2^ (Figure 5, inset), indicating rather slow and localized dynamics. Therefore, in the outer perovskite layer, even jumps between adjacent sites on the same octahedron are severely restricted below 1000 °C. Only at 1200 °C and above, can a change in the behavior of O3 and O4 oxygen atoms (Figure 5, inset) be observed, with atoms on the O3 site moving considerably further. However, the MSD remains approximately 1 order of magnitude lower than that of O1 and O2 oxygen atoms.

To get additional insights into the contribution to long-range diffusion from oxygen atoms located on different crystallographic sites, we have identified and analyzed all oxygen jumps taking place during the simulations. A jump is defined as an absolute displacement of more than 1 Å in either a, b, or c direction for at least 5 ps, to avoid counting random fluctuations. Table 1 summarizes the ionic hopping frequency, calculated as the number of jumps observed per ns.

**Table 1:** Ionic Hopping Frequency per Oxygen Site in ns^–1^

				(*ab*)/*c* (ns^–1^)						
along	600 °C		800 °C		1000 °C		1200 °C		1400 °C	
O1	3	13	23	97	77	267	163	303	197	443
O2	0	30	17	127	37	413	57	457	110	663
O3	0	10	0	30	0	23	3	73	13	227
O4	0	10	0	7	0	17	0	43	0	100

**Figure 6:**
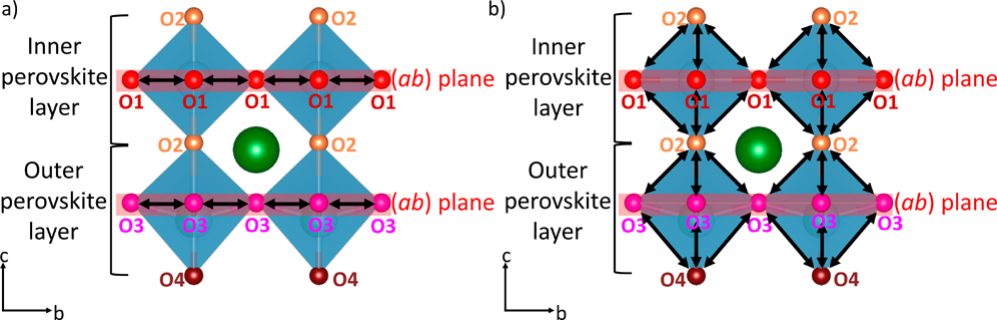
Schematic visualization of possible oxide ion jumps in CsBi_2_Ti_2_NbO_10−δ_ in the (*ab*) plane (*a*) and out-of-plane (b).

As before, oxygen atoms are separated according to the site at which they are initially located. In addition, observed jumps are separated into two categories: jumps in the (*ab)* plane, and jumps along the *c* axis (Figure 6), distinguishing in-plane O1–O1 and O3–O3 jumps (Figure 6a), from out-of-plane O1–O2, O2–O3, and O3–O4 jumps (Figure 6b).

**Figure 7:**
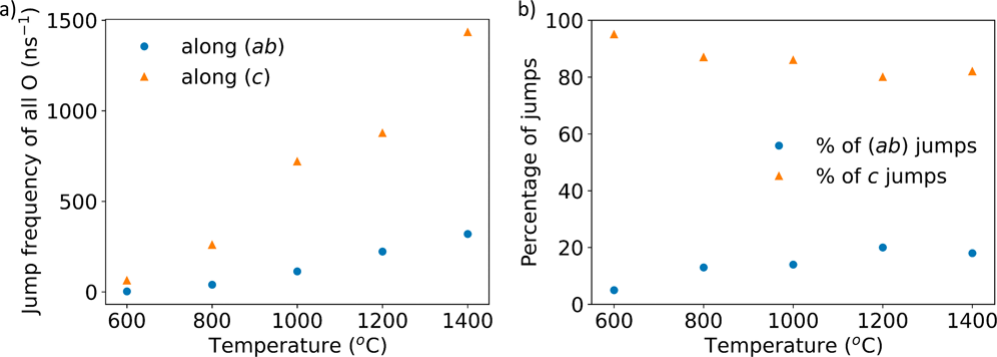
Ionic hopping frequency of all O sites in the (*ab*) plane and along (*c*) at increasing temperatures.

Figure 7 shows a visualization of the number of observed jumps in the (*ab*) plane and along c, highlighting that at all simulated temperatures out-of-plane jumps are significantly more frequent. At 1200 and 1400 °C, jumps in the (*ab*) plane could be observed from all oxygen sites except O4 (Table 1). However, the number of jumps of oxygen atoms on the O3 site is significantly lower than that of O1 or O2 oxygen atoms. It should be noted that all O2 oxygen atoms must jump to an O1 site along *c* before in-plane diffusion of O2 oxygen atoms can occur. Because of the considerable distance between neighboring O2 sites (3.9 Å), no direct O2–O2 jumps take place. The simulations emphasize that, at all temperatures, oxygen atoms originating at the O1 and O2 sites are highly mobile, while those on O3 and O4 sites jump significantly less frequently.

**Figure 8:**
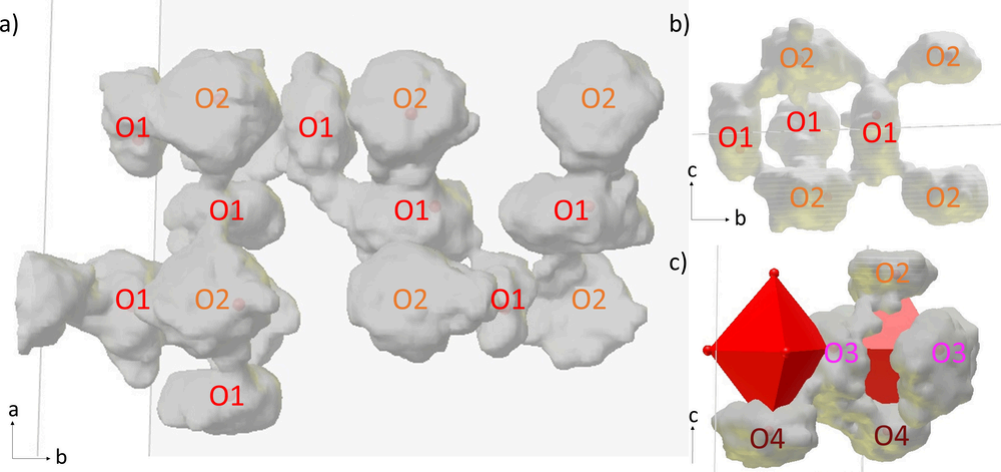
Cloud plots showing the region visited by selected oxygen atoms during the 300 ps AIMD trajectory at 1000 °C. They reveal a continuous O1–O2–O1 pathway (a, b) and show an example of jumps between O2–O3 and O3–O4 (c).

For the visualization of oxide ion diffusion, the trajectory obtained at 1000 °C was used as the large number of jumps at 1200 and 1400 °C (Table 1) made distinguishing separate pathways difficult. Cloud plots are shown in Figure 8, highlighting the paths followed by the oxygen atoms during the simulation. The presence of continuous O3–O4–O3–O4 and O2–O3–O4 paths, shown in Figure 8c, demonstrates that jumps are possible from all four distinct oxygen sites within the crystal structure. Nevertheless, the small MSD observed for oxygen atoms on O3 and O4 sites (Figure 5b) indicates that their total contribution to long-range diffusion is minor.

Most notably, the simulations show that the O2 sites play a major part in long-range diffusion by creating a continuous O1–O2–O1 diffusion pathway, as shown in Figure 8a,b, while direct jumps between O1 sites are less frequent. This directly observed diffusion pathway combined with the observation that, for O1 oxygen atoms, the number of out-of-plane jumps is substantially larger than the number of jumps in the (*ab*) plane (Table 1), highlights that it is more probable for oxygen atoms on O1 sites to move out-of-plane to an O2 site than to a neighboring O1 site.

It has been suggested^4^ that Bi^3+^ cations are displaced along the *c* and *a* (= *b*) axis because of the large Cs^+^ cations. This was proposed to result in more favorable bottlenecks for O1–O1 and O1–O2 oxide ion diffusion. Our AIMD simulations indicate that such displacement affects O1–O2 jumps more favorably than O1–O1 jumps.

The strong correlation of simulated MSD curves for oxygen atoms on O1 and O2 sites (Figure 5a), the observed diffusion pathways (Figure 8a,b), and the larger number of jumps along *c*, out-of-plane, counted at all simulated temperatures (Table 1) indicates that the O1–O2–O1 pathway is the major contribution to the long-range oxide ion diffusion.

## 4 Conclusions

We have used long *ab initio* molecular dynamics simulations at five different temperatures to investigate the oxide ion dynamics in the Dion–Jacobson phase oxide ion conductor CsBi_2_Ti_2_NbO_10−δ_.

Our results offer crucial new insight into oxide ion dynamics in CsBi_2_Ti_2_NbO_10−δ_. We demonstrated that, regardless of the starting distribution of oxide ion vacancies, the oxygen atoms at O1 and O2 sites exhibit the largest mobility, and while some O3 and O4 oxygen dynamics were observed, their contribution to long-range diffusion is limited. A detailed analysis of oxide ion jumps revealed that diffusion does not predominantly occur via O1–O1 jumps in the (*ab*) plane, but instead that jumps perpendicular to this plane are significantly more favorable. This indicates that the dominant oxide ion diffusion pathway is through O1–O2–O1 jumps, suggesting that ionic conductivity in CsBi_2_Ti_2_NbO_10−δ_ could be further improved by promoting jumps perpendicular to the (*ab*) plane. This can be achieved through an increase of the rotational flexibility of the octahedral units. For this, vanadium doping is a viable option, as it is known that VO_*x*_ groups can facilitate polyhedral rotation, and V^5+^ can support 4, 5, and 6 coordinate environments.^37^ This could also increase oxide ion mobility along O3–O2–O3 sites, further improving the oxide ion conductivity in CsBi_2_Ti_2_NbO_10−δ_.
